# ﻿A new skink of the genus *Scincella* Mittleman, 1950 (Squamata, Scincidae) from Son La Province, northwestern Vietnam

**DOI:** 10.3897/zookeys.1226.139655

**Published:** 2025-02-10

**Authors:** Anh Van Pham, Thomas Ziegler, Cuong The Pham, Thao Ngoc Hoang, Hanh Thi Ngo, Minh Duc Le

**Affiliations:** 1 Faculty of Environmental Sciences, University of Science, Vietnam National University, Hanoi, 334 Nguyen Trai Road, 11400 Hanoi, Vietnam; 2 Cologne Zoo, Riehler Straße 173, 50735, Cologne, Germany; 3 Institute of Zoology, University of Cologne, Zülpicher Straße 47b, 50674, Cologne, Germany; 4 Institute of Ecology and Biological Resources, Vietnam Academy of Science and Technology, 18 Hoang Quoc Viet Road, Hanoi, Vietnam; 5 Graduate University of Science and Technology, Vietnam Academy of Science and Technology, 18 Hoang Quoc Viet Road, Cau Giay, Hanoi, Vietnam; 6 Hong Duc University, Thanh Hoa, Thanh Hoa Province, Vietnam; 7 Central Institute for Natural Resources and Environmental Studies, Vietnam National University, Hanoi, 19 Le Thanh Tong, Hanoi, Vietnam; 8 Department of Herpetology, American Museum of Natural History, Central Park West at 79th Street, New York 10024, USA

**Keywords:** COI, molecular phylogeny, morphology, *Scincellatruongi* sp. nov., Sop Cop Nature Reserve, taxonomy

## Abstract

A new species of the genus *Scincella* Mittleman, 1950 is described from northern Vietnam based on morphological and molecular evidence. *Scincellatruongi***sp. nov.** is characterized by a combination of the following characters: size medium (SVL up to 59.4 mm); primary temporals 2; external ear opening without lobules; loreals two; supralabials seven or eight; infralabials six or seven; nuchals in three pairs; midbody scales in 28 rows; dorsal scales smooth, in six rows across the back; paravertebral scales 60–67, not widened; ventral scales in 60–70 rows; ten smooth lamellae beneath finger IV and 13–15 beneath toe IV; toes not reaching to fingers when limbs adpressed along body; dorsal surface of body and tail bronze brown with few black spots, a dark stripe running from nostril to eye and extending from posterior corner of eye along upper part of flank to the middle of the tail. In the phylogenetic analyses, the new species is recovered as an independent lineage with no clear sister taxon and at least 17.3% genetic divergence from other species in the genus based on a fragment of the mitochondrial COI gene.

## ﻿Introduction

Son La Province is located in northwestern Vietnam and covered with 40% or 439,592 ha of evergreen forest (The People’s Committee of Son La Province 2007). However, the biodiversity of this province is poorly studied, in particular reptiles and amphibians. In recent years, ten new species have been described from Son La, namely *Amolopstruongi* Pham, Pham, Ngo, Sung, Ziegler & Le, 2023 (Pham et al. 2023b); *Gracixalustruongi* Tran, Pham, Le, Nguyen, Ziegler & Pham, 2023 (Tran et al. 2023); *Tylototritonanguliceps* Le, Nguyen, Nishikawa, Nguyen, Pham, Matsui, Bernardes & Nguyen, 2015 (Le et al. 2015a); *T.pasmansi* Bernardes, Le, Nguyen, Pham, Pham, Nguyen & Ziegler, 2020 (Bernardes et al. 2020), *Cyrtodactylussonlaensis* Nguyen, Pham, Ziegler, Ngo & Le, 2017 (Nguyen et al. 2017); *C.taybacensis* Pham, Le, Ngo, Ziegler & Nguyen, 2019 (Pham et al. 2019); *Hemiphyllodactylusvanhoensis* Luu, Hoang, Ha, Grismer, Murdoch, Sitthivong, Phimpasone & Grismer, 2024 (Luu et al. 2024); *Achalinusquangi* Pham, Pham, Le, Ngo, Ong, Ziegler & Nguyen, 2023 (Pham et al. 2023a); *A.timi* Ziegler, Nguyen, Pham, Nguyen, Pham, Van Schingen, Nguyen & Le, 2019 (Ziegler et al. 2019); and *A.vanhoensis* Ha, Ziegler, Sy, Le, Nguyen & Luu, 2022 (Ha et al. 2022), and ten new country records have been added based on findings from Son La to the herpetofauna of Vietnam (Pham et al. 2014, 2016; Le et al. 2015b; Nguyen et al. 2015b).

The genus *Scincella* Mittleman, 1950 currently contains 39 recognized species with a wide distribution in Asia and America (Uetz et al. 2024). It is characterized by having a lower eyelid with an opaque window (Smith 1935; Taylor 1963; Greer 1974; Ouboter 1986; Nguyen et al. 2010b); supranasals absent, hindlimbs pentadactyl, lamellae under the basal digits in one row (Nguyen et al. 2010b), and lower secondary temporal overlapped by an upper scale (Greer and Shea 2003; Nguyen et al. 2011).

In Vietnam, Nguyen et al. (2009) recorded three species of *Scincella*, viz. *S.doriae* (Boulenger), *S.melanosticta* (Boulenger), and *S.reevesii* (Gray). Since then, a total of 15 species of the genus have been documented from the country (Uetz et al. 2024). During the last five years, three new species have been discovered, namely *S.badenensis* Nguyen, Nguyen, Nguyen & Murphy, 2019 from Tay Ninh Province; *S.baraensis* Nguyen, Nguyen, Nguyen & Murphy, 2020 from Binh Phuoc Province; and *S.ouboteri* Pham, Pham, Le, Ngo, Ngo, Ziegler & Nguyen, 2024 from Hoa Binh Province (Nguyen et al. 2019, 2020; Pham et al. 2024).

**Figure 1.**
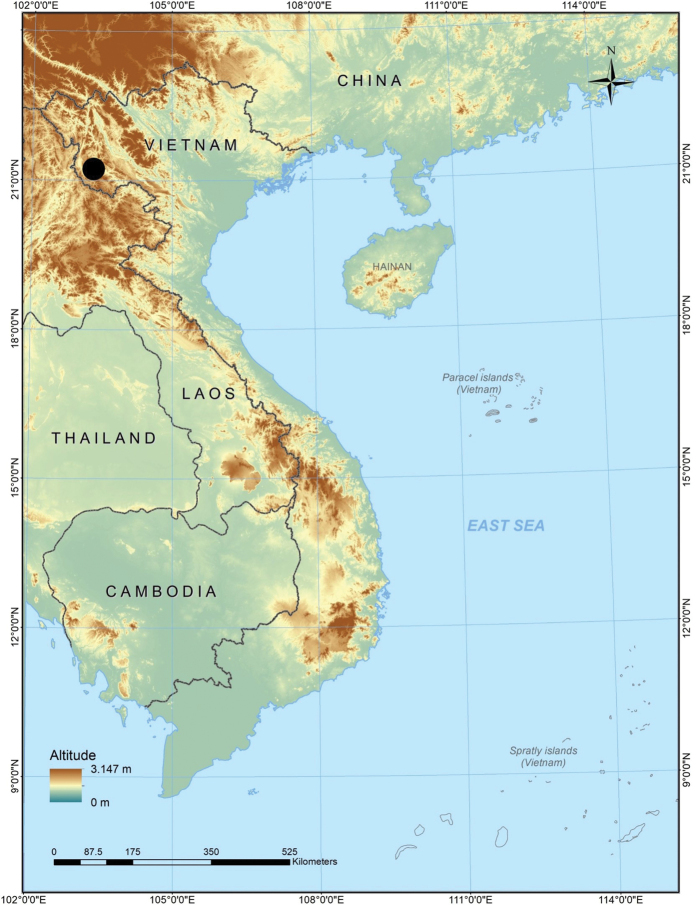
Map showing the type locality of *Scincellatruongi* sp. nov. in Son La Province, Vietnam

During our fieldwork in the evergreen forests of Sop Cop Commune, Sop Cop District, Son La Province, northwestern Vietnam, a new population of forest skinks was uncovered in Sop Cop Nature Reserve (Fig. 1). The collected specimens were assigned to the genus *Scincella* based on morphological examination. Further morphological and molecular analyses showed that they are distinctly differentiated from all other existing species. We therefore describe this population of *Scincella* from Son La Province as a new species herein.

## ﻿Materials and methods

### ﻿Sampling

A field survey was conducted in April 2013 in Sop Cop Nature Reserve, Son La Province, northwestern Vietnam. Specimens were collected between 8:00 and 16:00. After having been photographed in life, skinks were anesthetized and euthanized in a closed vessel with a piece of cotton wool containing ethyl acetate (Simmons 2002), fixed in 85% ethanol for ten hours, and then later transferred to 75% ethanol for permanent storage. Tissue samples were preserved separately in 70% ethanol before fixation. Voucher specimens were deposited in the collections of the University of Science (**HUS**), Vietnam National University, Hanoi (**VNU**) and the Institute of Ecology and Biological Resources (**IEBR**), Vietnam Academy of Science and Technology, Hanoi, Vietnam.

### ﻿Molecular data and phylogenetic analyses

We sequenced two samples of the new population from Son La Province. Additionally, ten ingroup and one outgroup taxa were included in the phylogenetic analysis following Pham et al. (2024) (Table 1). Tissue samples were extracted using DNeasy blood and tissue kit, Qiagen (Hilden, Germany). Extracted DNA from the fresh tissue was amplified by DreamTaq PCR mastermix (Thermo Fisher Scientific, Lithuania). A fragment of the mitochondrial cytochrome c oxidase subunit I (COI) was sequenced using primer pair LCO1490 (5’-GGTCAACAAATCATAAA GATATTGG-3’) and HCO2198 (5’--TAAACTTCAGGGTGACCAAAAAATCA-3’) (Folmer et al. 1994). The PCR volume consisted of 21 μl (10 μl of mastermix, 5 μl of water, 2 μl of each primer at 10 pmol/μl, and 2 μl of DNA or higher depending on the quantity of DNA in the final extraction solution). PCR condition was 95 °C for 5 min to activate the taq; with 40 cycles at 95 °C for 30 s, 50 °C for 45 s, 72 °C for 60 s; and the final extension at 72 °C for 6 min. PCR products were subjected to electrophoresis through a 1% agarose gel, 1^st^ BASE (Selangor, Malaysia). Gels were stained for 10 min in 1X TBE buffer at 2 pg/ml of ethidium-bromide and visualized under UV light. Successful amplifications were purified to eliminate PCR components using GeneJET™ PCR Purification Kit (Thermo Fisher Scientific, Lithuania). Purified PCR products were sent to Macrogen Inc. (Seoul, South Korea) for sequencing. Sequences generated in this study were edited using Geneious v. 7.1.8 (Kearse et al. 2012).

**Table 1.** Uncorrected (“p”) distance matrix showing percentage pairwise genetic divergence (COI) between the new species (highlighted in bold) and closely related species.

	Species	1	2	3	4	5	6	7	8	9	10	11	12	13	14	15	16	17	18	19	20	21	22
1	MG935701 *Sphenomorphusmaculata*	–																					
2	MH119607 *Scincellareevesii*	23.08	–																				
3	MH119609 *Scincellareevesii*	23.43	0.46	–																			
4	MH119611 *Scincellarufocaudata*	21.23	8.12	8.42	–																		
5	MH119612 *Scincellarufocaudata*	22.58	8.88	8.88	2.91	–																	
6	MH119613*Scincella* sp.	19.65	15.47	15.62	13.02	13.17	–																
7	MH119616 *Scincelladoriae*	20.18	21.26	21.27	19.22	19.37	17.27	–															
8	MH119617 *Scincelladoriae*	20.34	21.10	21.11	19.22	19.37	17.91	0.92	–														
9	MH119619 *Scincellamelanosticta*	23.80	18.38	18.38	19.45	18.99	19.60	18.71	18.87	–													
10	MH119621 *Scincellamelanosticta*	23.16	18.07	18.07	18.84	18.68	19.60	18.40	18.55	0.92	–												
11	MH119625 *Scincellarupicola*	21.02	20.74	21.06	18.54	19.02	16.61	20.14	20.14	21.50	21.67	–											
12	MH119627 *Scincellarupicola*	22.38	20.73	20.89	19.00	19.48	16.92	19.99	19.68	21.49	21.66	3.98	–										
13	MK990602 *Scincellabadenensis*	21.30	14.59	14.75	12.58	12.58	11.01	17.82	17.96	19.53	19.23	17.74	17.88	–									
14	MK990603 *Scincellabadenensis*	21.30	14.59	14.75	12.58	12.58	11.01	17.82	17.96	19.53	19.23	17.74	17.88	0.00	–								
15	MK990605 *Scincellanigrofascia*	19.57	16.78	16.63	14.62	14.62	3.53	16.42	16.72	20.33	20.33	16.80	16.80	9.91	9.91	–							
16	MT742256 *Scincellabaraensis*	23.12	20.13	20.13	19.65	19.81	18.86	16.13	16.28	18.91	19.07	20.46	21.50	18.47	18.47	17.87	–						
17	MT742257 *Scincellabaraensis*	23.12	20.13	20.13	19.65	19.81	18.86	16.13	16.28	18.91	19.07	20.46	21.50	18.47	18.47	17.87	0.00	–					
18	OP927028 *Scincellaochracea*	21.28	21.64	21.80	19.78	20.55	19.75	22.77	22.00	20.88	21.64	21.92	21.91	19.80	19.80	19.67	20.10	20.10	–				
**19**	**PQ666442*Scincellatruongi* sp. nov. HUS.2024.01**	**22.53**	**19.76**	**19.76**	**17.91**	**17.30**	**18.21**	**18.27**	**17.95**	**21.29**	**21.30**	**20.93**	**22.15**	**17.57**	**17.57**	**18.18**	**20.72**	**20.72**	**22.01**	–			
**20**	**PQ666443*Scincellatruongi* sp. nov. IEBR R.6329**	**22.53**	**19.76**	**19.76**	**17.91**	**17.30**	**18.21**	**18.27**	**17.95**	**21.29**	**21.30**	**20.93**	**22.15**	**17.57**	**17.57**	**18.18**	**20.72**	**20.72**	**22.01**	**0.00**	–		
21	OP927026 *Scincellaouboteri*	19.74	21.03	20.88	19.02	19.95	19.31	21.37	20.76	21.65	21.80	21.12	21.73	19.34	19.34	18.76	19.80	19.80	8.68	**21.11**	**21.11**	–	
22	OP927027 *Scincellaouboteri*	19.91	21.03	20.88	19.02	19.79	19.31	21.07	20.46	21.34	21.80	20.82	21.43	19.04	19.04	18.76	19.65	19.65	8.38	**20.81**	**20.81**	0.30	–

After sequences were aligned by Clustal X v. 2 (Thompson et al. 1997), data were analyzed using maximum parsimony (MP), as implemented in PAUP*4.0b10 (Swofford 2001), and Bayesian inference (BI), as implemented in MrBayes v. 3.2.7 (Ronquist et al. 2012). For MP analysis, heuristic analysis was conducted with 100 random taxon addition replicates using tree-bisection and reconnection (TBR) branch swapping algorithm, with no upper limit set for the maximum number of trees saved. Bootstrap support was calculated using 1000 pseudo-replicates and 100 random taxon addition replicates. All characters were equally weighted and unordered. For the maximum likelihood (ML) analysis, we used IQ-TREE v. 2.3.4 (Nguyen et al. 2015a) with a single model and 10,000 ultrafast bootstrap replications. The optimal model for nucleotide evolution was determined using jModeltest v. 2.1.4 (Darriba et al. 2012).

For Bayesian analyses, we used the optimal model selected by jModeltest with parameters estimated by MrBayes v. 3.2.7a. Two independent analyses with four Markov chains (one cold and three heated) were run simultaneously for ten million generations with a random starting tree and sampled every 1000 generations. Log-likelihood scores of sample points were plotted against generation time to determine stationarity of Markov chains. Trees generated before log-likelihood scores reached stationarity were discarded from the final analyses using the burn-in function. The posterior probability values for all clades in the final majority rule consensus tree were provided. The optimal model for nucleotide evolution was set to GTR+I+G for ML and single-model Bayesian analyses as selected by jModeltest v. 2.1.4. The cutoff point for the burn-in function was set to 25% of generated trees. Nodal support was also evaluated using bootstrap replication (BP) as estimated in PAUP, ultrafast bootstrap (UBP) in IQ-TREE v. 2.3.4, and posterior probabilities (PP) in MrBayes v. 3.2.7. BP ≥ 70, PP and UBP ≥ 95 were regarded as strong support for a clade (Hillis and Bull 1993; Ronquist et al. 2012; Nguyen et al. 2015a). Uncorrected pairwise divergences were calculated in PAUP*4.0b10.

### ﻿Morphological examination

Measurements were taken with a digital caliper (Electronic Digital Caliper) to the nearest 0.1 mm. The following morphological characteristics were recorded (after Nguyen et al. 2010a; Grismer and Quah 2015; Pham et al. 2024):

**SVL** snout-vent length (from tip of snout to cloaca);

**TaL** tail length (from cloaca to tip of tail);

**AG** distance from posterior junction of forelimb and body wall to anterior junction of hindlimb and body wall (with the limbs held at right angles to the body);

**HL** head length (from tip of snout to posterior margin of parietal or interparietal, depending on the longest distance);

**HW** head width (at the widest portion of temporal region);

**HH** head height (at the deepest portion of temporal region);

**SL** snout length (from anterior margin of eye to tip of snout);

**STL** distance from snout to anterior border of tympanum;

**SFlL** snout-forelimb length (from tip of snout to anterior junction of forelimb and body wall to the tip of fourth finger, with the limb held at right angles to the body);

**END** distance from anterior margin of eye to posterior border of nostril;

**EL** eye length (distance between anterior and posterior corners of eyelid);

**WIN** Window length (distance between anterior and posterior corners of window)

**TYD** maximum diameter of tympanum;

**FlL** forelimb length (from anterior junction of forelimb and body wall to the tip of fourth finger, with the limb held at right angles to the body);

**HlL** hindlimb length (anterior junction of hindlimb and body wall to the tip of fourth finger, with the limb held at right angles to the body).

### ﻿Scalation

Paravertebral scales counted as the number of scales in a line from posterior edge of parietals to dorsal point opposite posterior margin of the medial precloacals; transverse ventral scale rows counted as the number of scales from first gular scale to anterior margin of precloacals, number of subdigital lamellae under fourth finger and fourth toe. Bilateral scale counts were given as left/right. Sex identification was performed by inspection of presence or absence of hemipenes.

Morphological comparisons were based on data from the following literature: Gray (1838), Boulenger (1887), Günther (1896), van Denburgh (1912), Stejneger (1925), Barbour (1927), Schmidt (1927), Smith (1935), Taylor (1963), Zhao and Huang (1982), Darevsky and Nguyen (1983), Ouboter (1986), Wang and Zhao (1986), Inger et al. (1990), Darevsky and Orlov (1997), Chen et al. (2001), Darevsky et al. (2004), Bourret (2009), Nguyen et al. (2010a, b, c, 2011), Pham et al. (2015, 2024), Neang et al. (2018), Nguyen et al. (2019, 2020), Koizumi et al. (2022), and Jia et al. (2023, 2024).

## ﻿Results

### ﻿Phylogenetic analyses

**Figure 2.**
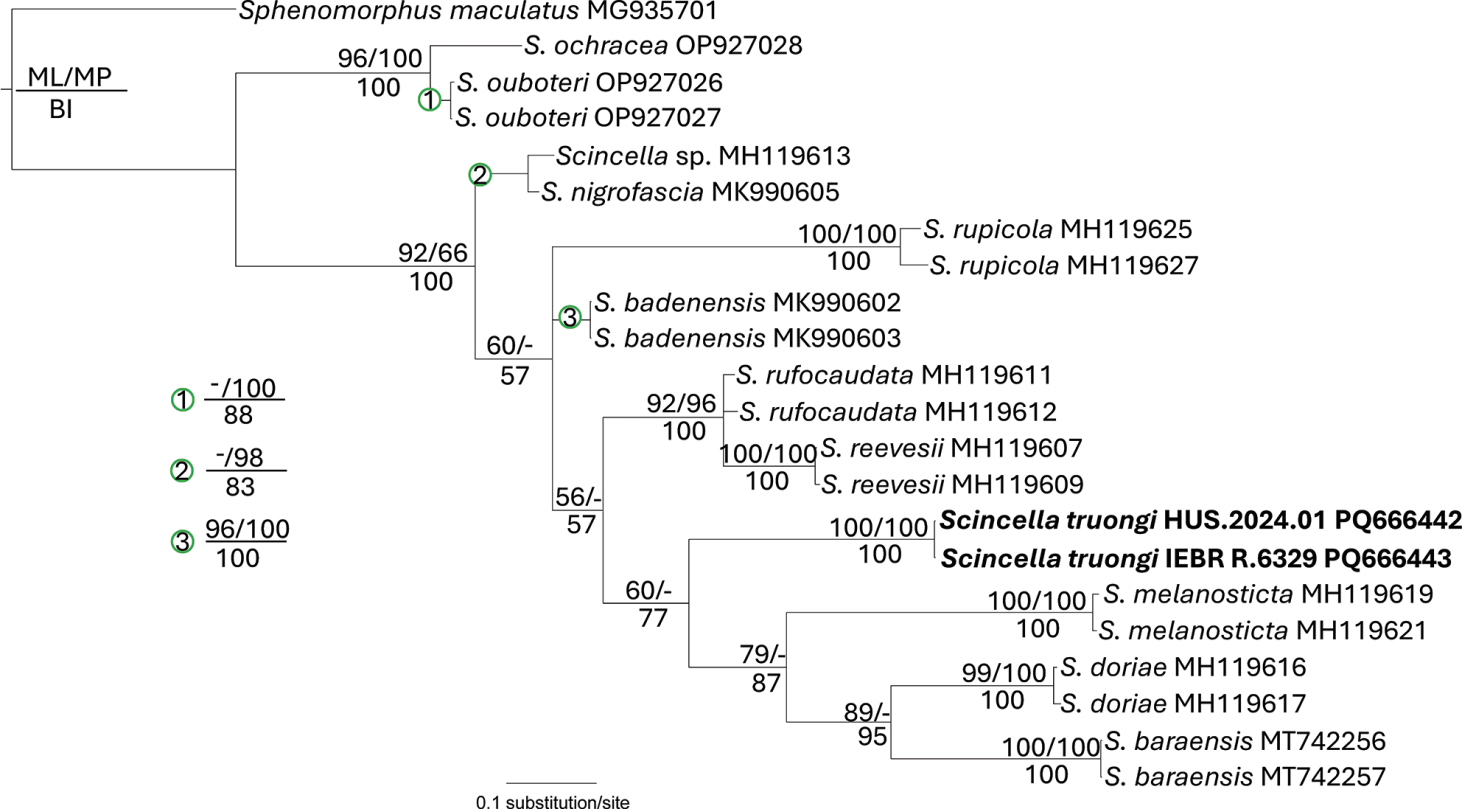
Phylogram based on the Bayesian analysis. Numbers above and below branches are ML ultrafast bootstrap/MP bootstrap values and single-model Bayesian posterior probabilities (> 50%), respectively. Dash indicates unsupported node. Letters and numbers after species names are GenBank accession records.

The aligned matrix of the molecular data contained 668 characters with no gaps, of which 247 were parsimony informative. The MP analysis produced two most parsimonious trees (Tree length = 864, Consistency index = 0.42, Retention index = 0.63). The new species from Son La Province was weakly placed as the sister taxon to a clade consisting of *S.baraensis* + *S.doriae* + *S.melanosticta* (BP < 50, UBP = 60, PP = 77) (Fig. 2). The three species are distributed either in southern Vietnam or broadly in the region. In terms of genetic divergences, the new species is separated from *S.baraensis*, *S.doriae* and *S.melanosticta* by 20.72%, 17.95–18.27%, and 21.29–21.30%, respectively. Genetically, it differs from the remaining species of *Scincella* included in the study by at least an uncorrected pairwise sequence divergence of ~17.3% (from *S.rufocaudata*) based on a fragment of the mitochondrial COI gene (Table 2).

**Table 2.** Morphological characteristics of *Scincellatruongi* sp. nov. from Son La Province, Vietnam.

	IEBR R.6329	IEBR R.6330	HUS. 2024.01	HUS. 2024.02	HUS. 2024.04	HUS. 2024.03	Min-Max
Type status	Holotype	Paratype	Paratype	Paratype	Paratype	Paratype	
Sex	Female	Male	Female	Female	Female	Female	
**Measurements** (in mm)							
SVL	54.86	49	51.66	54.01	59.4	53.7	49.0–59.4
TaL (*generated)	35*	32.8*	39.6*	36.1*	100.8	91.8	91.8–100.8
AG	32.15	25.29	29.4	31.15	34.3	31.4	25.3–34.3
SL	3.6	4	3.86	4	4.2	3.9	3.6–4.2
STL	9.8	9.98	9.38	10	9.98	9.3	9.3–10
SFlL	18.35	17.86	18.2	19.35	19.52	18.1	17.9–19.5
END	2.73	2.7	2.43	2.43	2.38	2.4	2.4–2.7
EL	3.11	2.9	2.72	3.2	3.24	2.7	2.7–3.2
HL	9.1	9.64	8.94	9.6	9.61	8.8	8.8–9.6
HW	6.57	6.19	5.89	7.1	7.05	6.6	5.9–7.1
HH	4.76	4.68	4.76	5	5.5	5.5	4.7–5.5
TYD	1.5	1.32	1.39	1.37	1.43	1.3	1.3–1.5
FlL	12.83	13	13.43	13.64	13	12.3	12.3–13.6
HlL	18.3	17.9	19.63	19.11	17.34	18.3	17.3–19.6
WIN	1.07	0.99	0.99	0.98	0.94	0.98	0.94–1.07
**Scalation**							
Supraoculars	4	4	4	4	4	4	4
Nuchals	3	3	3	3	3	3	3
Loreals	2	2	2	2	2	2	2
Preocular	1	1	1	1	1	1	1
Presuboculars	2	2	2	2	2	2	2
Supraciliaries	7	7	7	7	7	7	7
Postoculars	3	3	3	3	3	3	3
Postsuboculars	2	2	2	2	2	2	2
Primary temporals	2	2	2	2	2	2	2
Secondary temporals	2	2	2	2	2	2	2
Supralabials (R/L)	7	7/8	7	7	7	7	7–8
**Auricular lobules**							
Infralabials	7	7	7	7	6	7	6–7
Chin shields (pairs)	3	3	3	3	3	3	3
Midbody scale rows	28	28	28	28	28	28	28
Paravertebral scales	67	60	65	63	66	65	60–67
Ventrals in transverse rows	68	60	64	70	64	65	60–70
Precloacals (enlarged)	2	2	2	2	2	2	2
Lamellae on finger IV	10	10	10	10	10	10	10
Lamellae on toe IV	15	14	14	14	15	13	13–15

### ﻿Taxonomic account

#### ﻿ Scincella truongi sp. nov.

Taxon classificationAnimaliaSquamataScincidae

C0225AAA-E788-51A0-ACF6-C1E1B2D183E9

https://zoobank.org/09E36087-F43A-4D13-8918-C6F3AD27881E

Figs 3 , 4

##### Material examined.

**Figure 3.**
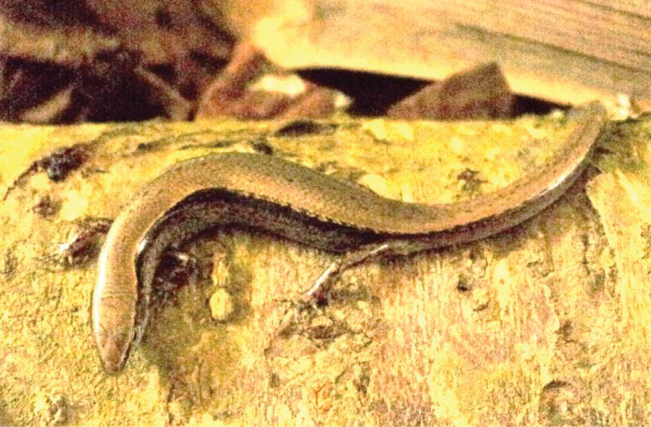
Holotype of *Scincellatruongi* sp. nov. (IEBR R.6329) in life, adult male. Photographs: AVP.

**Figure 4.**
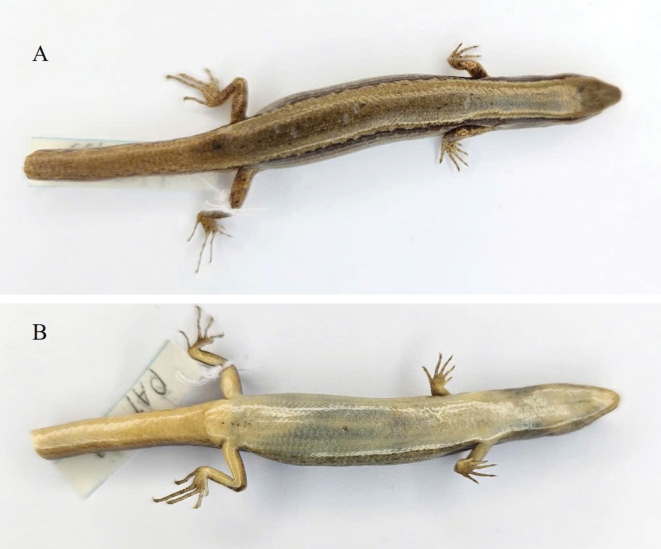
Holotype of *Scincellatruongi* sp. nov. (IEBR R.6329) in preservative **A** dorsal view **B** ventral view. Photographs: AVP.

**Figure 5.**
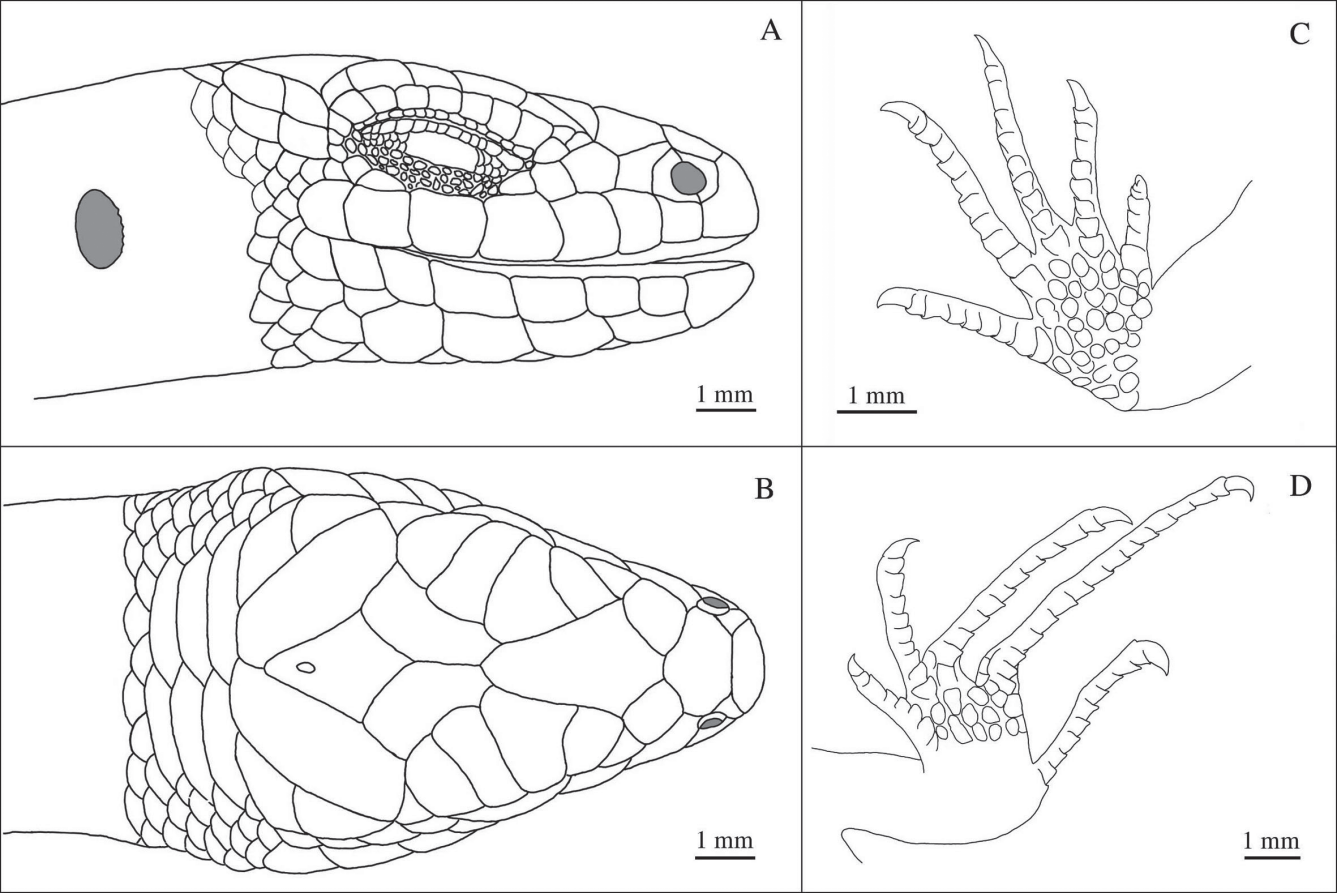
Holotype of *Scincellatruongi* sp. nov. (IEBR R.6329): Head **A** lateral view **B** dorsal view **C** hand **D** foot. Photographs: TNH.

**Figure 6.**
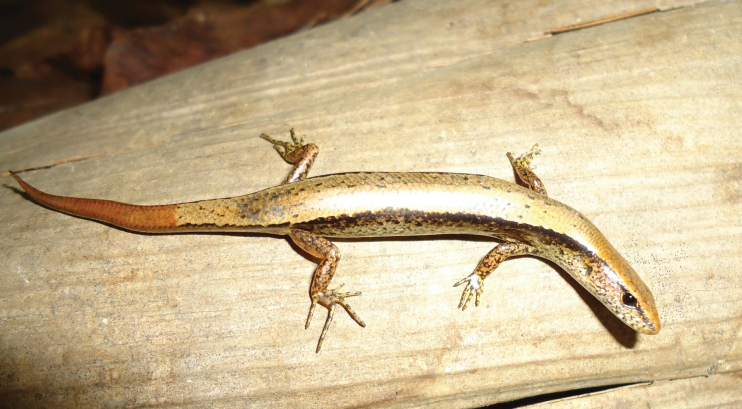
Paratype of *Scincellatruongi* sp. nov. (HUS.2024.01) in life, adult female. Photographs: AVP.

**Figure 7.**
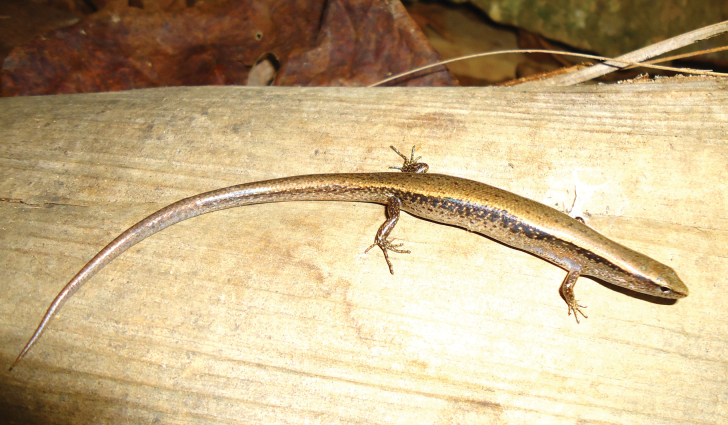
Paratype of *Scincellatruongi* sp. nov. (HUS.2024.03) in life, adult female. Photographs: AVP.

***Holotype*.** • IEBR R.6329 (Field number PAT.133) (Figs 3, 4A, B, 5A–D), adult female, collected on 3 April 2013 by A.V. Pham and D.A. Nguyen in evergreen forest near Ta Co Village (20°59'13.6"N, 103°37'19.4"E, at an elevation of 1660 m a.s.l.), Sop Cop Commune, within Sop Cop Nature Reserve, Sop Cop District, Son La Province, Vietnam. ***Paratypes*.** • IEBR R.6330 (Field number PAT. 129), adult male; HUS.2024.01 (PAT.130) (Fig. 6), adult female; HUS.2024.02 (PAT.131), adult female; HUS.2024.03 (PAT 134) (Fig. 7); and adult female, HUS.2024.04 (PAT. 164), adult female, bear the same data as the holotype.

##### Diagnosis.

The new species can be distinguished from other species of *Scincella* by a combination of the following characteristics: size medium (SVL up to 594 mm); primary temporals two; external ear opening without lobules; loreals two; supralabials seven or eight; infralabials six or seven; nuchals in three pairs; midbody scales in 28 rows; dorsal scales smooth, in six rows across the back; paravertebral scales 60–67, not widened; ventral scales in 60–70 rows; ten smooth lamellae beneath finger IV and 13–15 beneath toe IV; toes not reaching to fingers when limbs adpressed along body; dorsal surface of body and tail bronze brown with few black spots, a dark stripe running from nostril to eye and extending from posterior corner of eye along upper part of flank to the middle of the tail.

##### Description of holotype.

Size medium (SVL 54.86 mm), tail regenerated (TaL 35 mm); head longer than wide (HL 9.1 mm, HW 6.57 mm, SVL/HL 6.02); snout obtuse, round anteriorly; rostral wider than high, distinctly visible from above; supranasals absent; frontonasal wider than long, in contact with rostral, nasals, anterior loreals, and frontal; prefrontals not in contact with each other; frontal narrowing posteriorly, approximately 1.8 times longer than the distance to the tip of snout, in contact with prefrontals, first and second supraoculars, and frontoparietals; frontoparietals in contact with each other anteriorly, bordered by frontal, three supraoculars, parietals, and interparietal; interparietal lozenge-shaped, transparent spot posteriorly absent; parietals in contact posteriorly, posterolateral border surrounded by three scales on each side; nuchal scales in three pairs; nostril in center of nasal, in contact with rostral, frontonasal, loreal, first supralabial; loreals two, anterior loreal higher but narrower than posterior one; preocular one; presuboculars two, in contact with lower preocular, third and fourth supralabials; supraciliaries seven; supraoculars four, the first longest, the second widest, fourth supraocular followed by a small postsupraocular; postoculars three, anterior one in contact with seventh supraciliary, postsupraocular, and upper postsubocular; postsuboculars two, lower one in contact with sixth supralabial; primary temporals two, lower one in contact with sixth and seventh supralabials; secondary temporals two, upper very large, in contact with posterolateral, border of parietal, overlapped by lower one; lower eyelid with an undivided opaque window (central disc), separated from supralabials by two rows of granular scales; supralabials seven, fifth below the eye; external ear opening present, anterior margin with indistinct lobules, tympanum deeply sunk; mental wider than long, round anteriorly, in contact with anterior infralabials and postmental; infralabials seven, first small; postmental undivided, in contact with mental, first and anterior portion of second infralabials on each side, and first pair of chinshields; chinshields in three pairs, first pair in contact with each other medially, second pair separated from each other by a gular scale, and third pair separated from each other by three scales; midbody scales in 28 rows; dorsal scales smooth, in six rows across the back; paravertebral scales 67, not widened; ventral scales smooth, in 68 rows; precloacals four, inner scales overlapping outer ones, central two enlarged, left one overlapped by right one; tail thick at base, medial subcaudals widened vertical length of tail. Limbs relatively developed (SVL/FlL 4.28, SVL/HlL 3.0), pentadactyl, dorsal surface of digits covered by two scale rows on basal and by a single row on terminal phalanges; subdigital lamellae keeled, in one row under the digits, ten under fourth finger and 15 under fourth toe; toe and finger separated when adpressed along body, adpressed forelimb reaching to eye (Table 3).

**Table 3.** Morphological comparison between *Scincellatruongi* sp. nov. and two similar taxa from northwestern Vietnam.

Selected characters	*Scincellatruongi* sp. nov.	*S.ouboteri*	*S.ochracea*
	*n* = 6	*n* = 10	*n* = 15
SVL	49.0–59.4	40.9–58.6	42.3–51.0
TaL	91.8–100.8	52.9–76.9	62.3–75.0
AG	25.3–34.3	22–34.3	20.8–30.0
SL	3.6–4.2	3.5–4.3	2.4–3.4
STL	9.3–10	8.0–10.2	7.0–8.7
SFlL	17.9–19.5	14.9–20.1	12.4–16.2
END	2.4–2.7	2.1–2.6	1.8–2.2
EL	2.7–3.2	2.2–3.0	1.7–2.9
HL	8.8–9.6	7.8–9.5	6.9–8.2
HW	5.9–7.1	5.4–7.5	4.8–5.8
HH	4.7–5.5	4.0–5.3	3.6–4.4
TYD	1.3–1.5	1.5–2.0	1.3–1.6
FlL	12.3–13.6	9.9–13.5	7.6–10.4
HlL	17.3–19.6	16.4–19.5	13.9–16.6
**Scalation**			
Supraoculars	4	4	4
Nuchals	3	2–4	0–3
Loreals	2	2	2
Supraciliaries	7	7	7–8
Primary temporals	2	2	1
Secondary temporals	2	2	2
Supralabials (R/L)	7–8	7	7–8
Auricular lobules		3–4	3–4
Infralabials	6–7	6–7	5–6
Chin shields (pairs)	3	3	3
Midbody scale rows	28	32	30–32
Paravertebral scales	60–67	65–73	61–67
Ventrals in transverse rows	60–70	65–71	66–71
Precloacals (enlarged)	4	4	4–4
Lamellae on finger IV	10	10–12	9–11
Lamellae on toe IV	13–15	18–20	15–17

##### Coloration in life.

Dorsal surface of body and tail bronze brown with black spots; a dark stripe running from nostril to eye and extending from posterior margin of eye along upper part of flank and tail middle; lateral side of the head and flank with a few white spots; chin, throat and venter cream, outer edge of ventral scales dark grey; underside of fore and hind limbs lightly brown; ventral surface of tail greyish cream (Figs 3, 6, 7).

##### Variation.

IEBR R.6330 has 7/8 supralabials; infralabials 6/6 in paratype (HUS.2024.04); paravertebral scales 60 in IEBR R.6330, 65 in HUS.2024.01 and HUS.2024.03, 63 in HUS.2024.02, 66 in HUS.2024.04; ventrals in transverse rows 60 in IEBR R.6330, 64 in HUS.2024.01 and HUS.2024.04, 70 in HUS.2024.02, 65 in HUS.2024.03; lamellae on toe IV 14 in IEBR R.6330, 64 in HUS.2024.01 and HUS.2024.02, 15 in HUS.2024.04, 13 in HUS.2024.03.

##### Distribution.

*Scincellatruongi* sp. nov. is currently known only from the type locality in Son La Province, Vietnam (Fig. 1).

##### Natural history.

Specimens were found on the ground in leaf litter of evergreen forest during the daytime between 8:00 and 16:00. The surrounding habitat was evergreen forest with large, medium, and small hardwoods mixed with shrubs. Air temperatures at the sites ranged from 26.0–34.0 °C and relative humidity was 50–70%. Other reptile species encountered at the sites included *Acanthosauralepidogaster* (Cuvier), *Sphenomorphusindicus* (Gray), and *Pareashamptoni* (Boulenger).

##### Etymology.

We name the new species in honor of Prof. Dr. Truong Quang Nguyen from the Institute of Ecology and Biological Resources, Vietnam Academy of Science and Technology, in recognition of his great contributions to the herpetofaunal exploration of the Indochina region. We recommend “Truong’s Smooth Skink” as the common English name and “Thằn lằn cổ trường” as the common name in Vietnamese for the new species.

##### Comparisons.

We compared the new species with other known taxa in the genus *Scincella* from Asia based on examination of specimens and data obtained from the literature.

Morphologically, the new species resembles *Scincellaochracea* and *S.ouboteri* two other species known from northwestern Vietnam. However, they are distinguished from *S.ochracea* by having a larger size in the new species (males with maximum SVL 49.0 mm, *n* = 1 vs 45.4 mm, *n* = 7 and females with maximum SVL 59.4 mm, *n* = 4 vs 51.0 mm, *n* = 8), external ear opening without lobules (vs present), prefrontal in separate (vs contact), fewer midbody scale rows (28 vs 30–32), fewer lamellae beneath toe IV (13–15 vs 15–17), and different dorsal color pattern (dorsum with black spots vs with a dark discontinuous stripe) and from *S.ouboteri* by having fewer midbody scale rows (28 vs 30–32), fewer lamellae beneath toe IV (13–15 vs 18–20), toes separating fingers when limbs adpressed along body (vs overlap), and different dorsal color pattern (dorsum with black spots vs with two silver grey stripes, wide dark stripes) (Table 3).

*Scincellatruongi* sp. nov. has two primary temporals and thus differs from the following species in the genus *Scincella*: *S.apraefrontalis*, *S.baraensis*, *S.darevskii*, *S.devorator*, *S.melanosticta*, *S.monticola*, *S.punctatolineata*, and *S.rara*, which have only one primary temporal. The new species has toes not reaching fingers when limbs being adpressed along body, which differs from the following species, where toes and fingers do overlap: *S.baraensis*, *S.badenensis*, *S.dunan*, *S.formosensis*, *S.macrotis*, *S.melanosticta*, *S.reevesii*, and *S.rupicola*. In addition, the new species has the external ear opening without lobules and thus differs from the following taxa (external ear opening with lobules): *S.boettgeri*, *S.darevskii*, *S.ouboteri*, and *S.reevesii*.

The new species differs from *S.apraefrontalis* by having more midbody scale rows (28 vs 18), more paravertebral scales (60–67 vs 52), more ventral scales (60–70 vs 50), dorsal scales not enlarged (vs distinctly enlarged), more lamellae beneath toe IV (13–15 vs 8 or 9), and the presence of prefrontal (vs absent); from *S.baraensis* by having fewer dorsal scale rows on back (6 vs 8), fewer midbody scale rows (28 vs 30), and fewer lamellae beneath toe IV (13–15 vs 18–20); from *S.badenensis* by having more nuchal pairs (3 vs 0–1), fewer midbody scale rows (28 vs 32–36), and fewer lamellae beneath toe IV (13–15 vs 18–20); from *S.barbouri* by having fewer paravertebral scale (60–67 vs 70–79) and fewer nuchal pairs (3 vs 4 or 5), and more supraciliaries (7 or 8 vs 5 or 6); from *S.boettgeri* by having more nuchal pairs (3 vs 2); from *S.capitanea* by having fewer midbody scale rows (28 vs 30–32) and smaller body size (49.0–59.4 mm vs 78.5 mm); from *S.darevskii* by having fewer lamellae beneath toe IV (13–15 vs 17) and fewer supraoculars (4 vs 5); from *S.devorator* by having fewer lamellae beneath toe IV (13–15 vs 17–19) and different dorsal color pattern (dorsum with black spots vs with two silver grey stripes, wide dark stripes); from *S.doriae* by having fewer nuchal pairs (3 vs 4 or 5) and fewer midbody scale rows (28 vs 30–32); from *S.dunan* by having prefrontal in separate (vs contact) and and different dorsal color pattern (dorsum with a few black spots vs with many black spots); from *S.huanrenensis* by having fewer ventral scales (60–70 vs 75–89); from *S.liangshanensis* by having different dorsal color pattern (dorsal surface bronze brown with black spots (vs light brown with central dark brown) and more midbody scale rows (28 vs 23–27); from *S.macrotis* by having more nuchal scales (3 pairs vs 2) and fewer midbody scale rows (28 vs 31 or 32); from *S.melanosticta* by the presence of nuchal scales (3 pairs vs absent), fewer lamellae beneath toe IV (13–15 vs 16–22), fewer midbody scale rows (28 vs 34–38), and fewer dorsal scale rows on back (6 vs 10); from *S.modesta* by having more supraciliaries (7 or 8 vs 5–7), upper margin of lateral longitudinal striation relatively straight (vs wavy); prefrontal in separate (vs contact), and different lateral color pattern (upper margin of lateral with a dark stripe vs lateral dark with light spots); from *S.monticola* by having more midbody scale rows (28 vs 22–26), more paravertebral scales (60–67 vs 52–59), more dorsal scale rows on back (6 vs 4), and more ventral scales (60–70 vs 52–58); from *S.nigrofasciata* by having more nuchal scales (3 pairs vs 0 or 1), fewer midbody scale rows (28 vs 32–33), and fewer dorsal scale rows on back (6 vs 8); from *S.potanini* by having more midbody scale rows (28 vs 27) and fewer lamellae beneath toe IV (13–15 vs 17); from *S.przewalskii* by having more supralabials (7 vs 6), fewer midbody scale rows (28 vs 32–34), and fewer lamellae beneath toe IV (13–15 vs 17); from *S.punctatolineata* by having larger body size (SVL 49.0–59.4 mm vs 37.6–40.2 mm) and more nuchal scales (3 pairs vs 0–2); from *S.rara* by having more midbody scale rows (28 vs 24), more paravertebral scales (60–67 vs 53), and a single row of lamellae beneath toes II–IV (vs double rows); from *S.reevesii* by the presence of nuchals scales (3 pairs vs 0 or 1), fewer midbody scale rows (28 vs 29–35), and fewer dorsal scale rows on back (6 vs 8); from *S.rufocaudata* by the presence of nuchal scales (3 pairs vs absent), fewer dorsal scale rows on back (6 vs 10), and fewer midbody scale rows (28 vs 30–34); from *S.rupicola* by having fewer midbody scale rows (28 vs 34–36), fewer dorsal scale rows on back (6 vs 8), and the presence of nuchals scales (3 pairs vs 0 or 1); from *S.schmidti* by having more midbody scale rows (28 vs 26) and more lamellae beneath toe IV (13–15 vs 11); from *S.tsinlingensis* by having fewer paravertebral scales (60–67 vs 70–90) and fewer ventral scales (60–70 vs 70–90); from *S.vandenburghi* by having more lamellae beneath toe IV (13–15 vs 12) and upper margin of lateral longitudinal striation relatively straight (vs wavy); from *S.victoriana* by having more midbody scale rows (28 vs 26) and dorsal scales smooth (vs keeled); and from *S.wangyuezhaoi* by having fewer ventral scales (60–70 vs 71–89) and different dorsal color pattern (bronze-brown with black spots vs dark bronze-brown).

## ﻿Discussion

Over the last five years, six additional species have been described within the genus *Scincella* (Uetz et al. 2024). Three of the species, i.e., *S.badenensis*, *S.baraensis*, and *S.ouboteri*, were newly discovered and recorded in Vietnam (Nguyen et al. 2019, 2020; Pham et al. 2024). Our discovery brings the number of *Scincella* species in Son La Province to three, and in Vietnam to 16. In terms of morphology, *S.truongi* closely resembles the distantly related *S.ochracea* and *S.ouboteri* but the new species differs from *S.ochracea* by having a larger body size, external ear opening without lobules, separated prefrontal, fewer midbody scale rows, fewer lamellae beneath toe IV, and different dorsal color pattern and from *S.ouboteri* by having fewer midbody scale rows, fewer lamellae beneath toe IV, toes not reaching fingers when limbs being adpressed along body, and different dorsal color pattern. In the phylogenetic tree, the new species was recovered as an independent lineage with no clear sister taxon.

The new species is currently only found in Sop Cop Nature Reserve, a protected area established in 2002 in Son La Province. Although the new species has a small known range with an estimate of less than 50 km^2^, which has been experiencing severe habitat degradation primarily as a result of road construction and timber logging it is unclear whether these activities will significantly threaten its population, but it likely adversely affects it. We recommend listing the species as Data Deficient based on the IUCN Red List categories and criteria. Further research is needed to clarify the population status of this species and to determine specific anthropogenic threats at the site.

## Supplementary Material

XML Treatment for Scincella truongi

## ﻿Additional information

### Author ORCIDs

Anh Van Pham https://orcid.org/0000-0002-6023-3418

Thomas Ziegler https://orcid.org/0000-0002-4797-609X

Cuong The Pham https://orcid.org/0000-0001-5158-4526

Thao Ngoc Hoang https://orcid.org/0000-0001-9305-5518

Hanh Thi Ngo https://orcid.org/0000-0002-5283-6243

Minh Duc Le https://orcid.org/0000-0002-2953-2815

### Data availability

All of the data that support the findings of this study are available in the main text.

## References

[B1] Barbour T (1927) A new lizard from China. Copeia 165: 95.

[B2] Bernardes M Le MD Nguyen TQ Pham CT Pham AV Nguyen TT Rödder D Bonkowski M Ziegler T (2020) Integrative taxonomy reveals three new taxa within the *Tylototritonasperrimus* complex (Caudata, Salamandridae) from Vietnam. ZooKeys 935: 121–164. 10.3897/zookeys.935.37138 32508505 PMC7256073

[B3] Boulenger GA (1887) An account of the reptiles and batrachians obtained in Tenasserim by M. L. Fea, of the Genoa Civic Museum. Annali del Museo Civico di Storia Naturale di Genova, Serie 2 5: 474–486.

[B4] Bourret R (2009) Les Lezards de L’Indochine. Edition Chimaira, Frankfurt am Main, 624 pp.

[B5] Chen SL Hikida T Han SH Shim JH Oh HS Ota H (2001) Taxonomic status of the Korean populations of the genus *Scincella* (Squamata: Scincidae). Journal of Herpetology 35(1): 122–129. 10.2307/1566034

[B6] Darevsky IS Nguyen SV (1983) New and little known lizard species from Vietnam. Zoologitchesky Zhurnal 62: 1827–1837. [in Russian]

[B7] Darevsky IS Orlov NL (1997) A new genus and species of scincid lizards from Vietnam: the first Asiatic skink with double rows of basal subdigital pads. Journal of Herpetology 31(3): 323–326. 10.2307/1565659

[B8] Darevsky IS Orlov NL Ho CT (2004) Two new lygosomine skinks of the genus *Sphenomorphus* Fitzinger, 1843 (Sauria, Scincidae) from northern Vietnam. Russian Journal of Herpetology 11(2): 111–120.

[B9] Darriba D Taboada GL Doallo R Posada D (2012) JModelTest 2: More models, new heuristics and parallel computing. Nature Methods 9: 772. 10.1038/nmeth.2109 PMC4594756 22847109

[B10] Folmer O Black M Hoeh W Lutz R Vrijenhoek R (1994) DNA primers for amplification of mitochondrial cytochrome c oxidase subunit I from diverse metazoan invertebrates. Molecular Marine Biology and Biotechnology 3(5): 294–299. 7881515

[B11] Gray JE (1838) Catalogue of the slender-tongued saurians, with descriptions of many new genera and species. Annals and Magazine of Natural History 2(10): 287–293. 10.1080/00222933809496676

[B12] Greer AE (1974) The generic relationships of the scincid lizard genus *Leiolopisma* and its relatives. Australian Journal of Zoology (Suppl. Ser. 31), 1–67. 10.1071/AJZS031

[B13] Greer AE Shea G (2003) Secondary temporal scale overlap pattern: A character of possible broad systematics Importance in Sphenomorphine skinks. Journal of Herpetology 37(3): 545–549. 10.1670/104-02N

[B14] Grismer LL Quah ESH (2015) The rediscovery of *Sphenomorphusmalayanus* Doria 1888 (Squamata: Scincidae) from the Titiwangsa Mountain Range of Peninsular Malaysia and its re-description as *S.senja* sp. nov. Zootaxa 3931(1): 63–70. 10.11646/zootaxa.3931.1.4 25781814

[B15] Günther A (1896) Report on the collections of reptiles, batrachians and fishes made by Messrs. Potanin and Berezowski in the Chinese provinces Kansu and Sze-chuen. Annuaire du Musee Zoologique de l’Academie des Sciences de St. Petersbourg 1: 199–219.

[B16] Ha NV Ziegler T Sy TD Le MD Nguyen TQ Luu VQ (2022) A new species of the genus *Achalinus* (Squamata: Xenodermidae) from Son La Province, Vietnam. Zootaxa 5168(3): 375–387. 10.11646/zootaxa.5168.3.8 36101279

[B17] Hillis DM Bull JJ (1993) An empirical test of bootstrapping as a method for assessing confidence in phylogenetic analysis. Systematics Biology 42(2): 182–92. 10.1093/sysbio/42.2.182

[B18] Inger RF Zhao EM Shaffer HB Wu G (1990) Report on a collection of amphibians and reptiles from Sichuan, China. Fieldiana: Zoology 58: 1–24. 10.5962/bhl.title.3126

[B19] Jia R Gao Z Huang J Ren J Jiang K Li D Li J (2023) A new species of the genus *Scincella* Mittleman, 1950 (Squamata: Scincidae) from Sichuan Province, Southwest China, with a diagnostic key of *Scincella* species in China. Asian Herpetological Research 14(1): 24–40. 10.16373/j.cnki.ahr.220054

[B20] Jia R Gao Z Wu D Ren J Jiang D Wu W (2024) A new species of the genus *Scincella* Mittleman, 1950 (Squamata: Scincidae) from Sichuan Province, Southwest China. Asian Herpetological Research 15(2): 115–129. 10.3724/ahr.2095-0357.2024.0016 PMC11759151 39858232

[B21] Kearse M Moir R Wilson A Stones-Havas S Cheung M Sturrock S Buxton S Cooper A Markowitz S Duran C Thierer T Ashton B Mentjes P Drummond A (2012) Geneious Basic: An integrated and extendable desktop software platform for the organization and analysis of sequence data. Bioinformatics 28(12): 1647–1649. 10.1093/bioinformatics/bts199 22543367 PMC3371832

[B22] Koizumi Y Ota H Hikida T (2022) A new species of the genus *Scincella* (Squamata: Scincidae) from Yonagunijima Island, Southern Ryukyus, Japan. Zootaxa 5128(1): 61–83. 10.11646/zootaxa.5128.1.3 36101186

[B23] Le DT Nguyen TT Nishikawa K Nguyen SLH Pham AV Matsui M Bernardes M Nguyen TQ (2015a) A new species of *Tylototriton* Anderson, 1871 (Amphibia: Salamandridae) from northern Indochina. Current Herpetology 34(1): 38–50. 10.5358/hsj.34.38

[B24] Le DT Pham AV Nguyen SLH Ziegler T Nguyen TQ (2015b) First Records of *Megophrysdaweimontis* Rao & Yang, 1997 and *Amolopsvitreus* (Bain, Stuart & Orlov, 2006) (Anura: Megophryidae, Ranidae) from Vietnam. Asian Herpetological Research 6(1): 66–72. 10.16373/j.cnki.ahr.140045

[B25] Luu VQ Hoang TT Ha HB Grismer LJ Murdoch M Sitthivong S Vilay P Grismer LL (2024) Integrative taxonomy reveals two new species of karst-dwelling *Hemiphyllodactylus* Bleeker, 1860 (Squamata: Gekkonidae) from the border region of Laos and Vietnam. Zootaxa 5486(1): 071–108. 10.11646/zootaxa.5486.1.3 39646841

[B26] Mittleman MB (1950) The generic status of *Scincuslateralis* Say, 1823. Herpetologica 6: 17–20.

[B27] Neang T Chan S Poyarkov NA (2018) A new species of smooth skink (Squamata: Scincidae: *Scincella*) from Cambodia. Zoological Research 39(3): 220–240. 10.24272/j.issn.2095-8137.2018.008 29683108 PMC5968863

[B28] Nguyen SV Ho CT Nguyen TQ (2009) Herpetofauna of Vietnam. Edition Chimaira, Frankfurt am Main.

[B29] Nguyen TQ Ananjeva NB Orlov NL Rybaltovsky E Böhme W (2010a) A new species of the genus *Scincella* Mittleman, 1950 (Squamata: Scincidae) from Vietnam. Russian Journal of Herpetology 17(4): 269–274.

[B30] Nguyen TQ Nguyen SV Böhme W Ziegler T (2010b) A new species of *Scincella* (Squamata: Scincidae) from Vietnam. Folia Zoologica 59(2): 115–121. 10.25225/fozo.v59.i2.a6.2010

[B31] Nguyen TQ Nguyen TT Böhme W Ziegler T (2010c) First record of the Mountain ground skink *Scincellamonticola* (Schmidt, 1925) (Squamata: Scincidae) from Vietnam. Russian Journal of Herpetology 17(1): 67–69.

[B32] Nguyen TQ Schmitz A Nguyen TT Orlov NL Böhme W Ziegler T (2011) Review of the genus *Sphenomorphus* Fitzinger, 1843 (Squamata: Sauria: Scincidae) in Vietnam, with description of a new species from northern Vietnam and southern China and the first record of *Sphenomorphusmimicus* Taylor, 1962 from Vietnam. Journal of Herpetology 45(2): 145–154. 10.1670/09-068.1

[B33] Nguyen LT Schmidt HA von Haeseler A Bui MQ (2015a) IQ-TREE: A fast and effective stochastic algorithm for estimating maximum-likelihood phylogenies. Molecular Biology and Evolution 32(1): 268–274. 10.1093/molbev/msu300 25371430 PMC4271533

[B34] Nguyen TQ Pham AV Nguyen SLH Le MD Ziegler T (2015b) First record of *Parafimbrioslao* Teynié, David, Lottier, Le, Vidal & Nguyen, 2015 (Squamata: Xenodermatidae) from Vietnam. Russian Journal of Herpetology 22(4): 297–300.

[B35] Nguyen TQ Pham AV Ziegler T Ngo HT Le MD (2017) A new species of *Cyrtodactylus* (Squamata: Gekkonidae) and the first record of C. otai from Son La Province, Vietnam. Zootaxa 4341(1): 025–040. 10.11646/zootaxa.4341.1.2 29245698

[B36] Nguyen SN Nguyen VDH Nguyen LT Murphy RW (2019) A new skink of the genus *Scincella* Mittleman, 1950 (Squamata: Scincidae) from Ba Den Mountain, Tay Ninh Province, southern Vietnam. Zootaxa 4648(2): 273–286. 10.11646/zootaxa.4648.2.4 31716948

[B37] Nguyen SN Nguyen VDH Nguyen LT Murphy RW (2020) A new skink of the genus *Scincella* Mittleman, 1950 (Squamata: Scincidae) from southern Vietnam. Zootaxa 4868(3): 423–434. 10.11646/zootaxa.4868.3.6 33311393

[B38] Ouboter PE (1986) A revision of the genus *Scincella* (Reptilia: Sauria: Scincidae) of Asia, with some notes on its evolution. Zoologische Verhandelingen 229: 1–66.

[B39] Pham AV Le DT Nguyen SLH Ziegler T Nguyen TQ (2014) First records of *Leptolalaxeos* Ohler, Wollenberg, Grosjean, Hendrix, Vences, Ziegler & Dubois, 2011 and *Hylaranacubitalis* (Smith, 1917) (Anura: Megophryidae, Ranidae) from Viet Nam. Russian Journal of Herpetology 21(3): 195–200.

[B40] Pham AV Le DT Nguyen SLH Ziegler T Nguyen TQ (2015) New provincial records of skinks (Squamata: Scincidae) from northwestern Vietnam. Biodiversity Data Journal 3: e4284. 10.3897/BDJ.3.e4284 PMC4319168 25698899

[B41] Pham AV Le DT Pham CT Nguyen SLH Ziegler T Nguyen TQ (2016) Two additional records of megophryid frogs, *Leptobrachiummasatakasatoi* Matsui, 2013 and *Leptolalaxminimus* (Taylor, 1962), for the herpetofauna of Vietnam. Revue suisse de Zoologie 123(1): 35–43. 10.5281/zenodo.46287

[B42] Pham AV Le MD Ngo HT Ziegler T Nguyen TQ (2019) A new species of *Cyrtodactylus* (Squamata: Gekkonidae) from northwestern Vietnam. Zootaxa 4544(3): 360–380. 10.11646/zootaxa.4544.3.3 30647245

[B43] Pham AV Pham CT Le MD Ngo HT Ong AV Ziegler T Nguyen TQ (2023a) *Achalinusquangi*, a new odd-scaled snake species from Vietnam. Zootaxa 5270(1): 048–066. 10.11646/zootaxa.5270.1.2 37518178

[B44] Pham AV Pham CT Sung NB Ngo HT Ziegler T Le MD (2023b) A new species of *Amolops* (Anura: Ranidae) from Son La Province, northwestern Vietnam. Raffles Bulletin of Zoology 71: 51–69. 10.26107/RBZ-2023-0004

[B45] Pham AV Pham CT Le MD Ngo HN Ziegler T Nguyen TQ (2024) A new skink of the genus *Scincella* Mittleman, 1950 (Squamata: Scincidae) from Hoa Binh Province, northern Vietnam. Zootaxa 5428(1): 91–106. 10.11646/zootaxa.5428.1.4

[B46] Ronquist F Teslenko M van der Mark P Ayres DL Darling A Höhna S Larget B Liu L Suchard MA Huelsenbeck JP (2012) MrBayes 3.2: efficient Bayesian phylogenetic inference and model choice across a large model space. Systematic Biology 61(3): 539−542. 10.1093/sysbio/sys029 PMC3329765 22357727

[B47] Schmidt KP (1927) Notes on Chinese reptiles. Bulletin of the American Museum of Natural History 54: 467–551.

[B48] Simmons JE (2002) Herpetological collecting and collections management. Revised edition. Society for the Study of Amphibians and Reptiles. Herpetological Circular 31: 1–153.

[B49] Smith MA (1935) The Fauna of British India, including Ceylon and Burma. Reptilia and Amphibia. Vol. II. Sauria. Taylor and Francis, London, 440 pp.

[B50] Stejneger L (1925) Description of a new scincid lizard and a new burrowing from China. Journal of the Washington Academy of Sciences 15: 150–152.

[B51] Swofford DL (2001) PAUP*. Phylogenetic Analysis Using Parsimony (* and Other Methods), version 4. Sinauer Associates, Sunderland, Massachusetts.

[B52] Taylor EH (1963) The lizards of Thailand. University of Kansas Science Bulletin 44: 687–1077.

[B53] The People’s Committee of Son La Province (2007) Son La Province Portal. http://sonla.gov.vn/gioi-thieu [accessed July 2024]

[B54] Thompson JD Higgins DG Gibson TJ (1997) CLUSTAL W: improving the sensitivity of progressive multiple sequence alignment through sequence weighting, position-specific gap penalties and weight matrix choice. Nucleic Acids Research, 22(22): 4673–4680. 10.1093/nar/22.22.4673 PMC308517 7984417

[B55] Tran TT Pham AV Le MD Nguyen NH Ziegler T Pham CT (2023) A new species of *Gracixalus* (Anura, Rhacophoridae) from northwestern Vietnam. ZooKeys 1153: 15–35. 10.3897/zookeys.1153.93566 37234482 PMC10208806

[B56] Uetz P Freed P Hošek J [Eds] (2024) The Reptile Database. http://www.reptile-database.org/ [accessed 12 July 2024]

[B57] van Denburgh J (1912) Concerning certain species of reptiles and amphibians from China, Japan, the Loo Choo Islands, and Formosa. Proceedings of the California Academy of Science 4(3): 187–258.

[B58] Wang YZ Zhao EM (1986) Studies on Chinese species of *Scincella* (Scincidae, Sauria). Acta Herpetologica Sinica, 5: 267–277. [in Chinese]

[B59] Zhao EM Huang KC (1982) A survey of amphibians and reptiles in Liaoning Province. Acta Herpetologica Sinica 1: 1–23. [in Chinese]

[B60] Ziegler T Nguyen TQ Pham CT Nguyen TT Pham AV van Schingen M Nguyen TT Le MD (2019) Three new species of the snake genus *Achalinus* from Vietnam (Squamata: Xenodermatidae). Zootaxa 4590(2): 249–269. 10.11646/zootaxa.4590.2.3 31716093

